# Lipopolysaccharide Inhibits the Channel Activity of the P2X7 Receptor

**DOI:** 10.1155/2011/152625

**Published:** 2011-09-14

**Authors:** Elias Leiva-Salcedo, Claudio Coddou, Felipe E. Rodríguez, Antonello Penna, Ximena Lopez, Tanya Neira, Ricardo Fernández, Mónica Imarai, Miguel Rios, Jorge Escobar, Margarita Montoya, J. Pablo Huidobro-Toro, Alejandro Escobar, Claudio Acuña-Castillo

**Affiliations:** ^1^Centro Fondap de Estudios Moleculares de la Célula Instituto de Ciencias Biomédicas, Facultad de Medicina, Universidad de Chile, Chile; ^2^Section on Cellular Signaling, Program on Developmental Neuroscience (PDN), NICHD, Bethesda, Md, USA; ^3^Departamento de Biología, Facultad de Química y Biología y Centro de Biotecnología Acuícola, Universidad de Santiago de Chile (USACH), Chile; ^4^Facultad de Ciencias Biológicas y Facultad de Medicina, Universidad Andrés Bello, Chile; ^5^Instituto de Química, Facultad de Ciencias, Pontificia Universidad Católica de Valparaíso PUCV, Avenida Universidad 330, Curauma, Valparaíso, Chile; ^6^Centro de Regulación Celular y Patología J.V. Luco y Departamento de Fisiología, Facultad de Ciencias Biológicas, Pontificia Universidad Católica de Chile, Chile; ^7^Departamento de Ciencias Básicas y Comunitarias, Facultad de Odontología, Universidad de Chile, Chile; ^8^Emory Black, Facultad de Ciencias Médicas, Universidad de Santiago de Chile (USACH), Chile

## Abstract

The purinergic P2X7 receptor (P2X7R) plays an important role during the immune response, participating in several events such as cytokine release, apoptosis, and necrosis. The bacterial endotoxin lipopolysaccharide (LPS) is one of the strongest stimuli of the immune response, and it has been shown that P2X7R activation can modulate LPS-induced responses. Moreover, a C-terminal binding site for LPS has been proposed. In order to evaluate if LPS can directly modulate the activity of the P2X7R, we tested several signaling pathways associated with P2X7R activation in HEK293 cells that do not express the TLR-4 receptor. We found that LPS alone was unable to induce any P2X7R-related activity, suggesting that the P2X7R is not directly activated by the endotoxin. On the other hand, preapplication of LPS inhibited ATP-induced currents, intracellular calcium increase, and ethidium bromide uptake and had no effect on ERK activation in HEK293 cells. In splenocytes-derived T-regulatory cells, in which ATP-induced apoptosis is driven by the P2X7R, LPS inhibited ATP-induced apoptosis. Altogether, these results demonstrate that LPS modulates the activity of the P2X7R and suggest that this effect could be of physiological relevance.

## 1. Introduction

The P2X7 receptor (P2X7R) is a member of the family of nonselective cationic channels gated by extracellular ATP, termed P2X receptors. This receptor has unique properties with respect to other P2X members, like its low affinity for ATP and its ability to be activated in murine cells by ADP-ribosylation through ADP-ribosyltransferase (ARTs) and nicotinamide adenine dinucleotide (NAD) [1, 2]. Unlike other P2XRs, the P2X7R forms a macropore when exposed to continuous or high-level applications of ATP that allows the passage of 900-Da molecules (as Lucifer Yellow) which often induces cell death (reviewed in [3, 4]). This process seems to involve both dilation of the channel pore and opening of additional channels, such as pannexin-1 [5]. Although P2X7R expression has been detected in various tissues, including neurons, glial cells, fibroblasts, smooth muscle, and endothelial and epithelial cells (reviewed in [4]), it is in immune cells, such as monocytes, macrophages, dendritic cells, and T cells, where P2X7R function has been more extensively studied [6]. This receptor is a crucial player for induction of inflammatory responses *in vivo*. For instance, the use of P2X7R antagonists abolishes the development of inflammatory response to LPS. P2X7R knock-out mice show lower mortality and proinflammatory cytokine production under systemic LPS challenge than wild-type animals [7]. Molecular mechanisms associated with the P2X7R in inflammation are linked to inflammasome activity including a role in the induction, maturation, and delivery of IL-1*β* (reviewed in [8, 9]). Under several pathophysiology conditions, such as cell necrosis, *in vivo* inflammatory responses, and in tissue trauma, NAD, ATP, and metabolites are released and reach extracellular concentrations that can activate P2X7Rs and promote cell death [2]. Therefore, immune cells that express P2X7R respond during inflammatory events. In this context, molecules that can regulate P2X7R activity, such as LPS, could also have a key regulatory role during these events. Similar effects have been proposed for other P2X7R modulators like divalent cations [10, 11], endogenous lipids, and hydrophobic compounds. For example, lysophosphatidylcholine potentiates Ca^2+^ influx, apoptosis, and ERK activation in mouse microglial cells [12]; PIP_2_ induces a positive modulation in P2X7R currents [13]; BSA, 11-((5-dimethylaminonaphthalene-1-sulphonyl)amino) undecanoic acid (DAUDA) and arachidonic acid (AA) markedly increases BzATP potency and stimulates ethidium accumulation [14]. AA also induces a robust increase in ERK1/2 phosphorylation when coapplied with BzATP, whereas no differences are observed in other MAPKs [15]. Polymyxin B and specifically its hydrophobic tail positively modulate P2X7R-mediated ethidium uptake [16]. Similarly, a diverse range of lipids increases agonist potency in the P2X7R in functional and binding studies [17]. P2X7R agonists alone can stimulate PLA2-mediated arachidonic acid release, and the antagonist OATP inhibits this effect [18]. In P388D1 murine macrophages, inhibition of the P2X7R attenuates LPS-stimulated prostaglandin production, which is correlated with reduced soluble PLA2 (sPLA2) activity and a reduction in the transcriptional upregulation of cyclooxygenase-2 [19]. Altogether, these findings suggest that P2X7R regulation by lipids could be relevant in inflammation. The idea that LPS modulates P2X7R activity was suggested by Denlinger et al. [20], who identified a conserved LPS-binding site in the C-terminal domain of the P2X7R. Synthetic peptides derived from this domain can bind LPS *in vitro*, leading to inhibition of LPS-induced ERK activation in RAW 264.7 cells [20].

Our aim was to determine whether LPS may modulate P2X7R activation and if it is related to physiological processes. We evaluated whether LPS can affect ATP-gated currents, intracellular calcium, ERK activation, and apoptosis in P2X7R-expressing HEK293 cells. This heterologous system does not express TLR-4, the main function of which is to identify the LPS of gram-negative bacterial cell wall and activate the innate immune system [21]. We found that LPS differentially modulates ATP-induced activity, inhibiting ATP-gated currents, intracellular calcium increases, and ethidium bromide uptake, while having no effect on ATP-dependent ERK activation. LPS treatment also inhibits ATP-induced apoptosis in CD4+ CD25+ regulatory T cells, protecting them from cell death. On the basis of these results, we propose that LPS may have a role as a regulator of P2X7R activity during inflammation.

## 2. Experimental Procedures

### 2.1. Animals, Cells, and Transfection of the P2X7 Receptor in HEK293 Cells

Male and female 6–8-week-old C57BL/6 mice were obtained from the USACH Research Facility, with their use approved by the Ethics Committee. Animals were euthanized by cervical dislocation, spleens were removed under sterile conditions, and splenocytes were obtained discarding erythrocytes by treatment with ACK buffer lyses. Splenocytes were cultured in RPMI 1640 medium (Invitrogen Corporation, Carlsbad, Calif, USA) supplemented with 10% FBS (Biological Industries Ltd., Kibbutz Haemek 25115, Israel), 50 U/mL penicillin-streptomycin, and 2.5 *μ*g/mL amphotericin B (Sigma-Aldrich, St. Louis, Mo, USA). All salts used were of analytical degree. Antibodies to CD4 and CD25 were purchased from Santa Cruz Biotechnology (clones RM-4 and PC61, resp.). Antibody to CD8 was obtained from BD Biosciences Pharmingen (San Diego, Calif). Regulatory T-cell kit detection assay was obtained from eBioscience (San Diego, Calif). Annexin V-FITC Apoptosis Detection Kit was purchased from BD Biosciences Pharmingen.

HEK293 and U937 cells were cultured in DMEM medium (Invitrogen Corporation, Carlsbad, Calif, USA) supplemented with 10% FBS (Biological Industries Ltd., Kibbutz Haemek 25115, Israel), 50 U/mL penicillin-streptomycin, and 2.5 *μ*g/mL amphotericin B (Sigma-Aldrich, St. Louis, Mo, USA). Rat P2X7R cloned into pIRES2-eGFP was kindly donated by Dr. S. Stojilkovic (NIH, Bethesda, Md, USA). Cells were transfected with lipofectamine (Invitrogen Corporation, Carlsbad, Calif, USA) following the manufacturer's instructions. Stable clones were isolated by G418 (EMD Biosciences, Inc, San Diego, Calif, USA) selection under standard procedures, and expression was evaluated routinely.

### 2.2. Electrophysiology

ATP-gated current was measured with an AXOPATCH 200B amplifier in whole cell configuration (Molecular Devices, Calif, USA), using standard patch-clamp techniques. The composition of the pipette solution was (mM): 140 KCl, 5 NaCl, 1 MgCl_2_, 10 EGTA, 10 HEPES, pH 7.2. The bath solution contained (mM): 140 NaCl, 5 KCl, 1 MgCl_2_, 1 CaCl_2_, 10 glucose, and 10 HEPES, pH 7.4. Recording pipettes had 4 MΩ of resistance. Command voltage protocols and acquisition were controlled by pCLAMP 10.2 (Molecular Devices, Calif, USA) through a laboratory interface (Digidata 1322A, Molecular Devices, USA). Unless otherwise shown, membrane holding potential (*V*_*m*_) was −80 mV. For ramp protocols, the holding potential was 0 mV, following a 1-s voltage ramp from −120 to +120 mV. Currents were filtered at 1 kHz with an eight-pole Bessel low-pass filter (Frequency Devices) and digitized at 5 kHz. The liquid junction potential between internal and external solutions was estimated to be 3.7 mV and was subtracted from all records. All the experiments were performed at room temperature; ATP was applied to the cells by a gravity perfusion system. Data were analyzed with Clampfit 10.2 software (Molecular Devices, Calif, USA). 

### 2.3. Intracellular Calcium Measurement

Intracellular calcium was measured with using the cell-permeable calcium indicator Indo-1 AM or Fura-2 AM. For Indo-transfected or control HEK293 cells were seeded at 10^5^ cells on 35 mm coverslips. The following day, the cells were incubated with 5 *μ*M Indo 1-AM in bath solution for 1.5 h in darkness. The cells were then washed twice with PBS and incubated in DMEM with serum for 1 h in darkness. Single cells were selected and excited at 360 nm, and fluorescence was detected with a photomultiplier at 405 and 488 nm (Nikon). Only one cell per coverslip was used for the experiment, and the bath solutions were the same as for electrophysiology. The calcium signal was obtained at a fluorescence ratio of 405/488 nm. 

### 2.4. Western Blots, RT-PCR, Ethidium Bromide Uptake, and Apoptosis

For Western blot analyses, P2X7R-HEK293 cells were seeded in 6-well plates or on 22-mm collagenized coverslips. The plates were stimulated with 600 *μ*M ATP (Sigma-Aldrich, St. Louis, Mo, USA), unless another concentration is indicated. LPS challenges were made using 0.1 *μ*g/mL LPS *E. coli* serotype 055 : B5 (Sigma-Aldrich, St. Louis, Mo, USA) incubated for different times. Treatments, methods, and antibodies used (except for anti-P2X7), and data acquisition were as previously described [22]. RNA extraction and RT-PCR and visualization of PCR products were done as previously described [23]. Amplification of 16S was done using specific primers (sense: 5′-GGGGTTTACGACCTCGATGTT-3′ antisense: 5′-GCTTTAAGTATGGGCCCCCCT-3′); PCR conditions were 94°C for 4 min followed by 29 cycles of 94°C for 40 s, 50°C for 40 s, and 72°C for 20 s. TLR-4 amplification was done using specific primers (sense CAACAAAGGTGGGAATGCTT 317, antisense TGCCATTGAAAGCAACTCTG), with PCR conditions 94°C for 4 min followed by 30 cycles of 98°C for 10 s, 60 for 2 min, 74 for 15 s.

To determine ethidium uptake, semiconfluent P2X7R-expressing HEK293 cells were incubated at 37°C in PBS nominally divalent cation-free buffer supplemented with 1% of FBS. After 10 min incubation, 20 *μ*g/mL ethidium bromide (Amresco Inc., Solon, Ohio) was added, and 1 min later, the cells were treated with ATP or LPS. The results were analyzed by confocal microscopy (LSM 510) using a Plan-Neofluar objective (20x; 0.5 NA; Carl Zeiss, Inc.) every 15 s for 30 min, with HeNe laser excitation with a 560-nm long-pass emission filter for ethidium bromide. Images were collected using LSM 510 software (Carl Zeiss, Inc.). 

To monitor induction of cell death, splenocytes were preincubated 30 minutes with or without LPS, Brilliant Blue G (Sigma-Aldrich, St. Louis, Mo, USA), or A740003 (*Tocris* Bioscience, Ellis-ville, Mo, USA) and challenged with 60 *μ*M ATP (Sigma-Aldrich) or, unless other conditions are specified, in RPMI 1640 supplemented with 10% FBS, 50 U/mL penicillin-streptomycin, and 2.5 *μ*g/mL amphotericin B, and cells were evaluated after 24 h of culture. Total CD4+ CD25+ and foxp3+ populations were evaluated at 24 h after challenge as previously described [24]. In parallel, cells were recovered 3 h after-treatment and stained to identify CD4+ and CD4+ plus CD25+ cells with the Annexin V-FITC Apoptosis Detection Kit I (BD Biosciences). Stained cells were detected in a FACS Canto II (Becton Dickinson, NJ USA).

### 2.5. Data Acquisition and Analysis

All protocols were performed at least three times on two clones. ATP-induced activation was evaluated in parallel with other protocols. Point-to-point analysis was performed using the Kruskal Wallis test, with a Dunn posttest. All data were analyzed using GraphPad software (GraphPad software Inc, La Jolla, Calif, USA) and are shown as average ± standard error (S.E.M). Statistical differences were considered with *P* < 0.05.

## 3. Results

### 3.1. Preapplied LPS Inhibits ATP-Evoked Currents in the Presence of FBS

**Figure 1:**
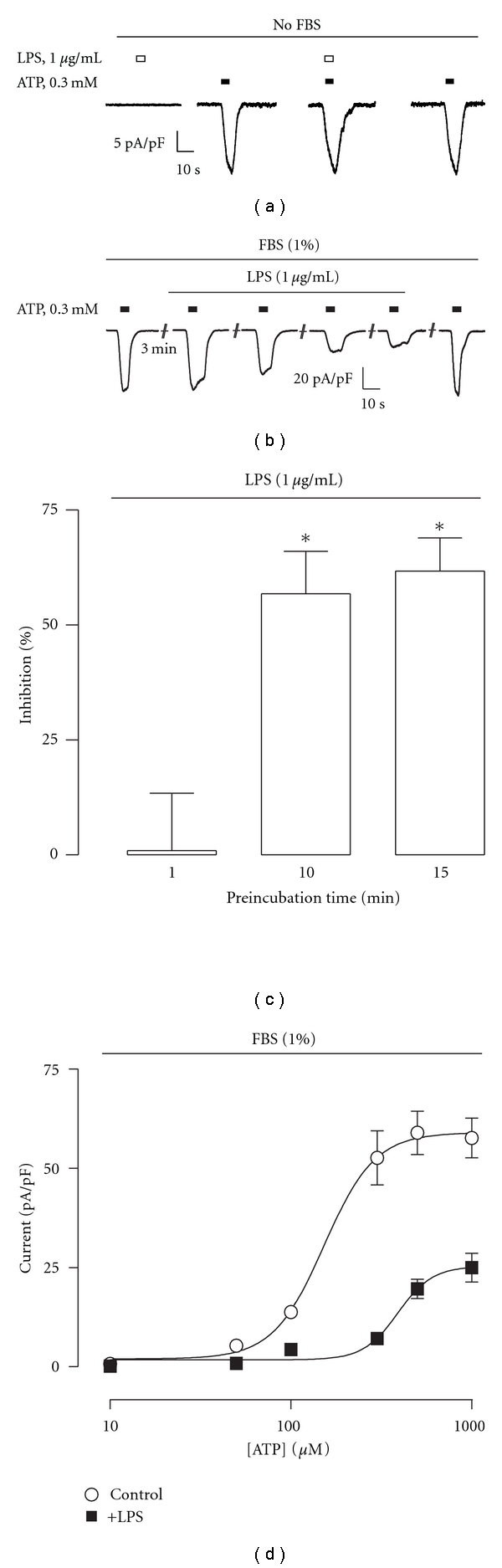
LPS inhibits ATP-induced currents in P2X7R-HEK293 cells. (a) LPS alone (1 *μ*g/mL) does not gate currents nor inhibit 300 *μ*M ATP-gated currents when coapplied. Data are representative of 5-6 independent experiments, in the absence of FBS. (b) Representative experiment of the LPS-induced inhibition of ATP-evoked currents. The effect of LPS is time-dependent, experiments were run in the presence of 1% FBS. (c) Summary of the effect of LPS on ATP-evoked currents after 1, 10, and 15 min of preapplication (*n* = 6–8, *P* < 0.05, Kruskal-Wallis test). (d) ATP concentration-response curves in the absence (open circles) or in the presence of 1 *μ*g/mL LPS (closed circles). LPS was added 30 min before the start of each experiment (*n* = 5).

**Figure 2:**
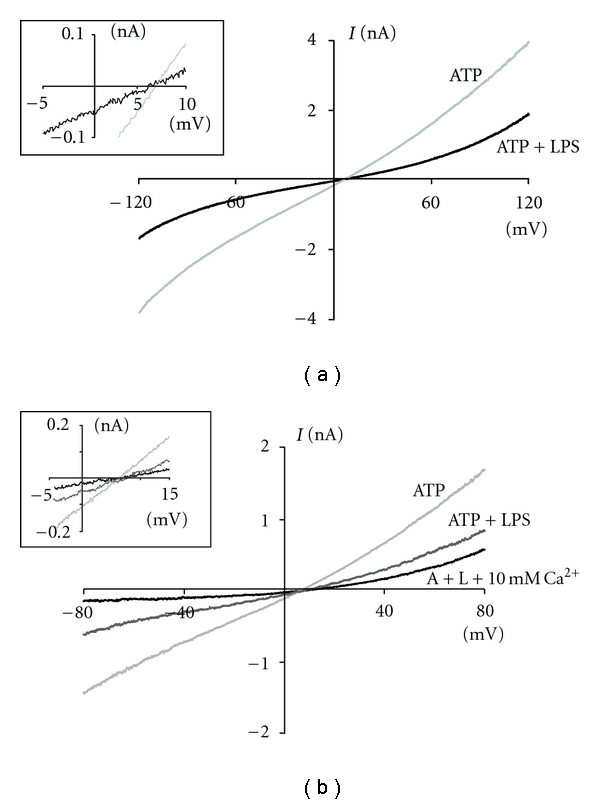
LPS does not change the permeability of the P2X7R. (a) Voltage-ramp protocols were applied with 300 *μ*M ATP alone (grey line) or treated with 1 *μ*g/mL LPS (black line); the insert shows a closeup of reversal potentials. Control and treated experiments were performed in parallel and repeated independently 4 times. (b) The same protocol was performed in the presence of 300 *μ*M ATP (ATP), preincubated with LPS (ATP + LPS) with normal and 10 mM extracellular calcium (A + L + 10 mM Ca^2+^).

To determinate whether LPS induces direct effects or modulates the activity of the P2X7R, we used HEK293 cells as an expression system. These cells do not express detectable levels of messenger levels by PCR nor protein on surface by flow cytometry of the canonical LPS receptor TLR-4 (see Figure S 1 in supplementary material available online at doi:10.1155/2011/152625). ATP evoked dose-dependent inward cationic currents in stable P2X7R-transfected HEK293 cells (P2X7R-HEK293), with an EC_50_ of 150 ± 58 *μ*M and a maximal current density of 18.4 ± 4.1 pA/pF (*n* = 6). As shown in Figure 1, LPS alone was unable to trigger currents (Figure 1(a)). Similarly, LPS coapplication (Figure 1(a)) or preapplications as long as 30 minutes (not shown) had no effect on ATP-induced currents. As a first screening of LPS-potential effects, a concentration of 300 *μ*M ATP was used, which is close to EC_50_, allowing us to determine either positive or negative effects. Since LPS-related effects have been shown to be dependent of serum factors like LPS-binding protein (LBP) [25], in subsequent experiments we evaluated the effects of LPS on ATP-induced currents, in the presence of 1% FBS in the extracellular recording solution. The addition of FBS increased the amplitude of ATP-induced currents but had no effect on ATP affinity (EC_50_ = 169 ± 9 *μ*M; *I*_max_ = 60 ± 6.1 pA/pF, *n* = 8). As shown in Figure 1(b), LPS reversibly inhibited the ATP-induced currents in a time-dependent manner. The inhibition of the ATP-evoked currents after 10 min of continuous LPS application was 57 ± 9% (Figure 1(c)). We then tested LPS effects along the entire ATP concentration response curve. LPS displaced rightward the curve, increasing ATP EC_50_ from 169 ± 9 to 375 ± 17 *μ*M (*P* < 0.01, *n* = 8; Figure 1(d)). LPS inhibited the ATP-induced response at all tested concentrations, reducing the maximal current density by 55%, from 60 ± 6.1 to 26 ± 3.9 pA/pF (*P* < 0.01, *n* = 8; Figure 1(d)). Next, we evaluated if LPS inhibition was related with changes in the reversal potential performing voltage ramps (Figure 2(a)). In order to minimize receptor desensitization during ramps, we used 300 *μ*M ATP (we used the same cells to perform ramps in the absence and presence of LPS). LPS decreased the ATP-induced currents at all voltages, with no changes in the reversal potential (*E*_rev_ = 6.8 ± 0.4 and 6.5 ± 0.2 mV in control and LPS-treated cells, resp., Figure 2(a), inset). This suggests that LPS treatment does not modify the permeability of the channel. To determine the potential role of Ca^2+^, its extracellular concentration was increased to 10 mM (Figure 2(b)). Under these conditions, we did not observe changes in reversal potential in cells treated with LPS (*E*_rev_ = 5.6 ± 0.8 mV; Figure 2(b), inset). Altogether, these results suggest that LPS modulates the ATP-dependent current when it is preapplied without modifying P2X7R ionic permeability.

### 3.2. P2X7R-Mediated Calcium Influx Is Inhibited by LPS Treatment

**Figure 3:**
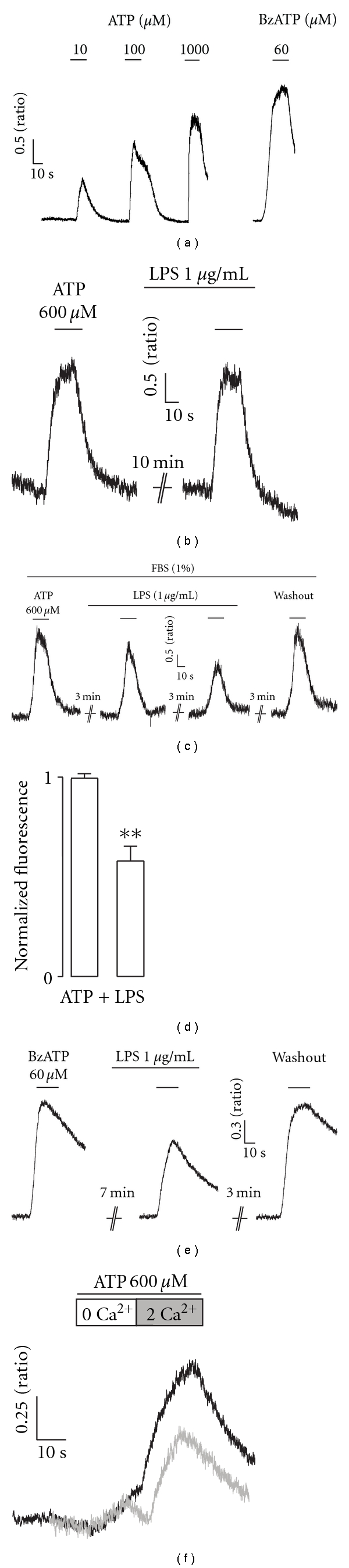
LPS inhibits ATP-induced intracellular calcium increases in P2X7R-HEK293 cells. (a) Representative tracings of a single cell responding to different ATP concentrations and to 60 *μ*M of the P2X7R-agonist BzATP. (b) Representative recordings showing the lack of effects of LPS in the absence of FBS. (c) Representative recordings showing the time dependent inhibition of ATP-induced calcium transients induced by 1 *μ*g/mL LPS, in the presence of 1% FBS. (d) Summary of the inhibition induced by 7 min of LPS preapplication (*P* < 0.01, *n* = 4). (e) Recordings of calcium increases induced by 60 *μ*M BzATP and inhibition by the pre-application of LPS. Similar results were observed in 3 different cells. (f) Recordings in the absence of extracellular calcium, in the absence (black tracing), and in the presence (gray tracing) of LPS (preapplied for 7 min).

Our next aim was to determine if the effects of LPS in P2X7R-mediated currents was also observed in P2X7R-mediated calcium influx. ATP induced a concentration-dependent increase of intracellular calcium in P2X7R-HEK293 cells (Figure 3(a)); this effect was mimicked by the specific P2X7R-agonist BzATP (Figure 3(a)). LPS-mediated effects were evaluated at a concentration that induces a maximal response (600 *μ*M) and that mostly desensitizes the potential responses by P2Y and adenosine receptors. As observed in current recordings, LPS had no effect on ATP-evoked responses when FBS was not present (Figure 3(b)). In the presence of 1% FBS, LPS inhibited calcium increases evoked by 600 *μ*M ATP (Figure 3(b)) in a time-dependent manner, in similar fashion to that observed in whole-cell currents (compare with Figure 1(b)). The inhibition of the ATP-evoked transients attained after 7 min of LPS application was 43 ± 9% (*P* < 0.01, *n* = 5; Figure 3(d)). In order to further discard the contribution of endogenous P2Y and adenosine receptors, we used the specific P2X7R-agonist Benzoyl-ATP (BzATP). A 7-min application of LPS-inhibited BzATP-induced calcium increases by 30% (Figure 3(e), *n* = 3). Finally, recordings performed in the absence of extracellular calcium confirmed that the LPS effects that we observed are specific to the P2X7R; the endogenous responses were minimal compared to the high calcium increases observed in HEK293-P2X7R cells (Figure 3(f) and Suppl. Figure  2).

### 3.3. LPS Inhibits ATP-Induced Macropore and Apoptosis

**Figure 4:**
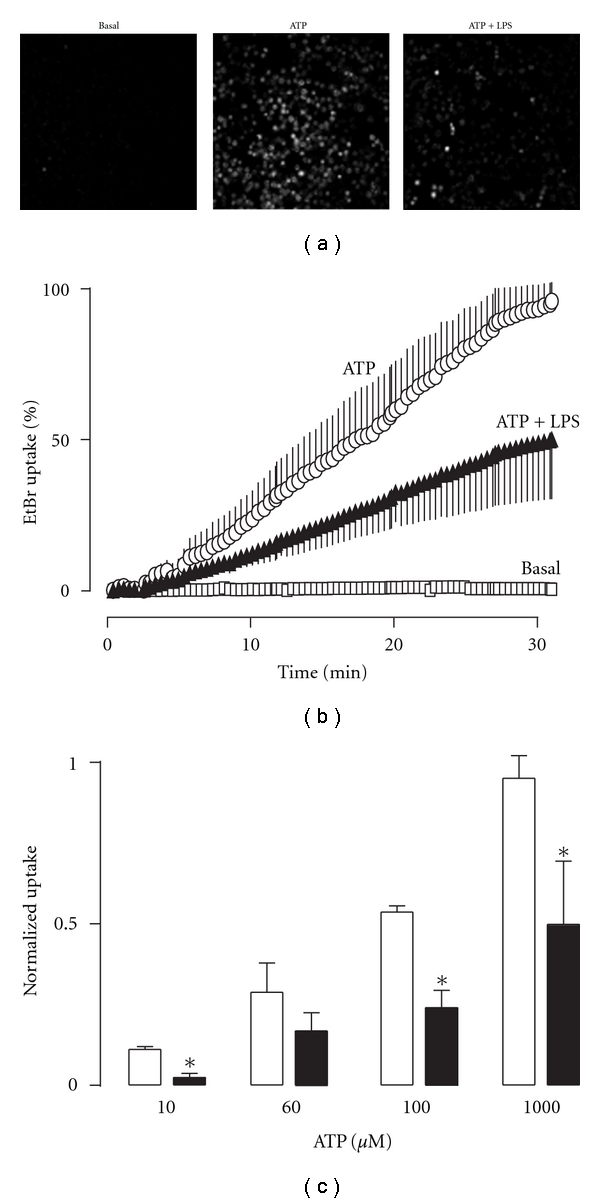
LPS inhibits the ATP-induced EtBr uptake on P2X7R-HEK293 cells. (a) Representative images obtained from EtBr uptake protocols in nontreated cells (basal) after 30 min of treatment with 1 mM ATP alone (ATP) or plus 1 *μ*g/mL LPS (ATP + LPS). (b) Summary of the time-dependent EtBr incorporation obtained with 1 mM ATP alone (open circles) or plus LPS (closed triangles); *n* = 4. (c) LPS inhibition of ATP-induced EtBr uptake at different ATP concentrations (*P* < 0.05, unpaired *t*-test; *n* = 3-4). Experiments were performed in the presence of 1% FBS.

We next evaluated if LPS could affect macropore formation. 1 mM ATP induced the uptake of ethidium bromide (EtBr) in the presence of 1% FBS. This concentration of ATP was chosen because it has been described as inducing maximal uptake. EtBr uptake was time-dependent, leading to maximum incorporation at 25–30 min (Figure 4(b)). In contrast, no staining was observed in control (Figure 4(a)) or in cells treated with LPS alone (not shown). However, LPS (preapplied for 30 min) significantly inhibited the ATP induced EtBt uptake (*P* < 0.05, Figures 4(a) and 4(b)). We next tested the effect of LPS at different ATP concentrations; Figure 4(c) shows that LPS consistently decreased EtBr uptake at 10, 60, 100, and 1000 *μ*M ATP (assays were made at 30 min after the ATP challenge).

**Figure 5:**
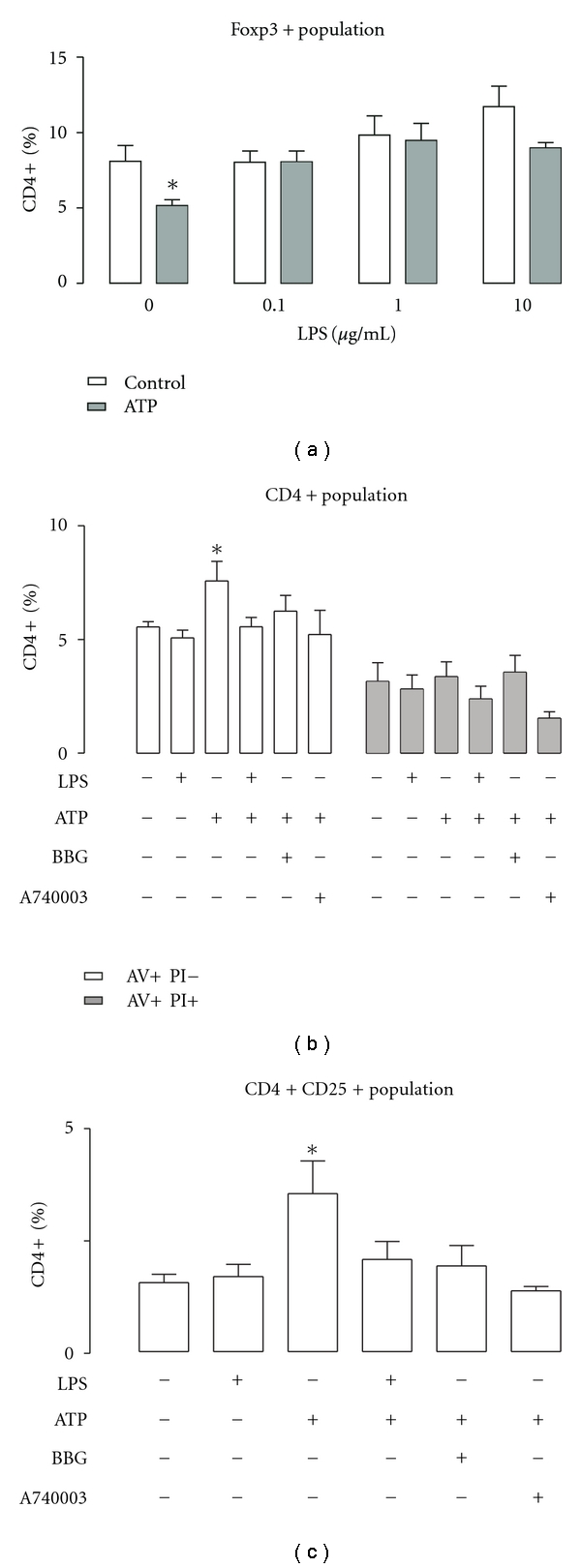
LPS protects from ATP-induced depletion and apoptosis in CD4+ CD25+ T-regulatory cells. (a) LPS abolishes the ATP-induced depletion of Treg's population. ATP (60 *μ*M, gray bars) was applied for 30 min, 24 h before cell staining and flow cytometry. *P < 0.05, Mann-Whitney test, *n* = 4. (b) Effects of LPS (1 *μ*g/mL) and BBG (100 nM, a P2X7R antagonist) in ATP-induced early apoptosis measured with annexin V (AV) assay. ATP (60 *μ*M) was applied for 30 min, 3 h before cell staining and flow cytometry. Open bars correspond to the apoptotic population, whereas gray bars represent necrotic cells. *P < 0.05, Mann-Whitney test, *n* = 4. (c) LPS and BBG inhibit the ATP-induced increase in CD4+ CD25+, annexin-positive population. ATP was applied for 30 min, 3 h before cell staining and flow cytometry. *P < 0.05, Mann-Whitney test, *n* = 4.

Because these results indirectly indicated that LPS inhibits P2X7R-mediated apoptosis, we next used annexin V (an apoptosis assay) to confirm this. We decided to evaluate apoptosis in cells that endogenously express P2X7R. To that end, we used CD4+ CD25+ Treg lymphocytes, regulators of the immune response derived from splenocytes. This cell type has been demonstrated to be more sensitive and to show higher affinity for ATP compared to other cells, including conventional CD4 and CD8 cells. ATP-mediated effects have been demonstrated to be dependent on the P2X7R and at concentrations as low as 60 *μ*M [24]. We first determined the effects of ATP and LPS on the total splenocyte population. Treg cell death was evaluated as depletion of Treg from total splenocyte culture, after a 24 h treatment with 60 *μ*M ATP, in the presence of FBS. ATP induced 45 ± 5% depletion of Treg cells (Figure 5(a)). LPS alone did not induce changes (not shown); however, LPS preapplied at all concentrations tested abolished ATP-induced depletion (Figure 5(a)). Splenocytes include a variety of cell types, including some that are LPS-sensitive, so our results cannot be related exclusively to P2X7R inhibition (e.g., the pleiotropic increase in T-CD4+, as shown in Figure 5(a): compare first and last columns). In order to discard nonspecific effects, we evaluated the effect of acute LPS application on annexin V (AV) exposure as a measure of P2X7R-mediated early apoptosis. Splenocytes were pretreated for 30 min with LPS and challenged with ATP for 3 h (in FBS-containing medium). After that, cells were stained against CD4+, propidium iodine (PI), and AV and evaluated by flow cytometry. ATP induced a 47 ± 10% increase in AV+ PI-population, indicating an increase in the number of apoptotic cells (Figure 5(b)). No changes were observed in double-positive population, indicating that this effect was related to apoptosis in the CD4+ population. LPS alone did not induce any change; however, it abolished the ATP-induced increase in the AV+ PI-population (Figure 5(b)). To further confirm the role of the P2X7R, we used the specific antagonists Brilliant Blue G (BBG) and A740003. These antagonists behaved similarly to LPS, inhibiting the ATP-induced increase in AV+ PI-population (Figure 5(b)). This pattern was also observed in the CD4+ CD25+ population (Figure 5(c)). Altogether, these results suggest that LPS is able to protect to Treg cells from P2X7R-mediated ATP-induced apoptosis.

### 3.4. LPS Does Not Modify the ATP-Induced ERK Activation

**Figure 6:**
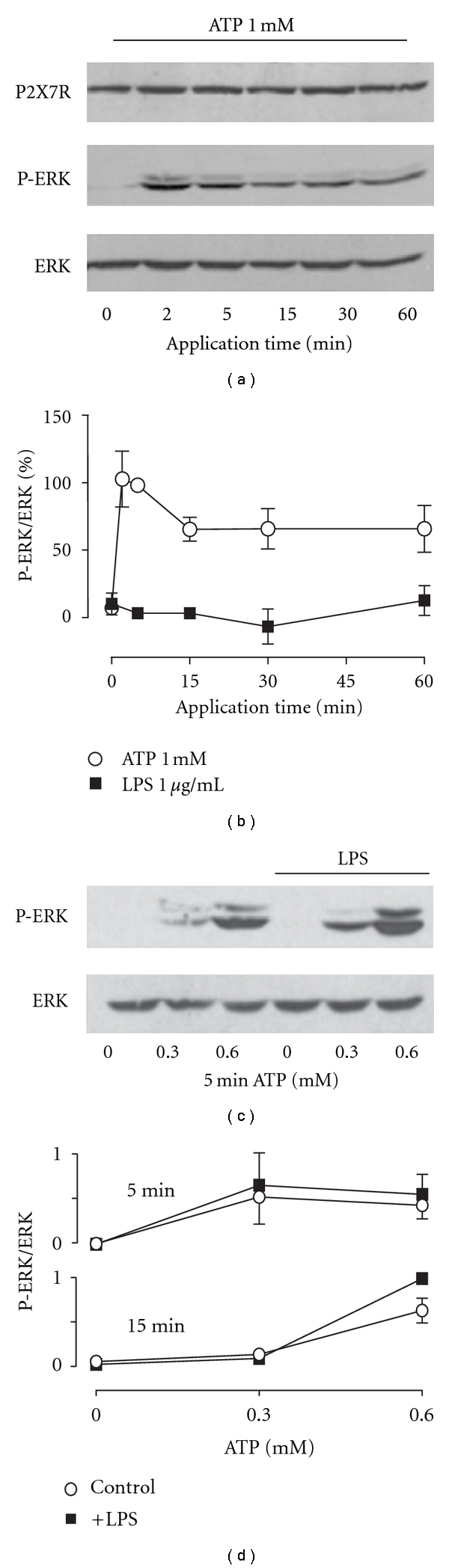
LPS does not affect ATP-induced ERK activation in P2X7R-HEK293 cells. (a) Representative gel showing ERK phosphorylation (P-ERK, middle panel) induced by 1 mM ATP applied at different times. The upper panel shows P2X7R immunoreactivity of stable transfected cells; the lower panel shows total ERK. (b) Quantification of the effects on ERK activation obtained by the application of 1 mM ATP (open circles) or 1 *μ*g/mL LPS (closed squares). Data were normalized against the phosphorylation induced by 2 min of ATP application, *n* = 6. (c) Representative gel showing control cells or pretreated with 1 *μ*g/mL LPS for 30 min. ATP was applied for 5 min, and ERK activation was evaluated. Upper panels show the detection of P-ERK; lower panels show total ERK. (d) Quantification of ERK activation in control (open circles) and LPS-treated (closed squares) cells at different ATP concentrations applied for 5 (upper graph) and 15 min (lower graph). Experiments were normalized against ERK phosphorylation induced by 600 *μ*M ATP for 15 min in LPS-treated cells.

Several signaling pathways are activated after P2X7R stimulation [26–30], including the ERK pathway. Our next aim was to determine if LPS-induced changes on P2X7R-mediated ERK activation, since it has been demonstrated that this effect is independent of its channel activity [31]. These authors described that in HEK293 cells, ATP induces ERK activation in P2X7R expression but not in mock cells [31]. In our case, we did not detect ATP-induced ERK activation in nontransfected HEK293 cells (data not shown), in agreement with Amstrup and Novak. In P2X7R-expressing cells, ATP induced a time-dependent activation of the ERK pathway (Figures 6(a) and 6(b)) in the presence of FBS, with a maximum activation of 2–5 min afterstimulation, decreasing later, and then remaining stable throughout the rest of the experiment (Figure 6(b)). Under similar conditions, LPS alone was unable to trigger this effect (Figure 1(b) and Supp. Figure  3), suggesting that it is not able to directly activate the ERK pathway in our cell system and further supporting the absence of endogenous TLR-4 receptors in HEK293 cells. As a control and to test LPS activity on the TLR-4 receptor, we observed a LPS activation of ERK pathway in the macrophage cell-line Raw 264.7 (Supp. Figure  3). Higher LPS concentrations (10 *μ*g/mL) also failed to induce ERK activation (data not shown). Next, we preapplied LPS for 30 min before ATP stimulation for 5 min on P2X7R-HEK293 cells. Under these conditions, LPS was unable to modify the ATP-induced changes in ERK activation (Figures 6(c) and 6(d)). Similarly, we did not observe differences when ATP was applied for 15 min (Figure 6(d)). Altogether, these results demonstrate that in our cell system, LPS does not act as an agonist of the ERK pathway or as a modulator of the ATP-induced ERK pathway.

## 4. Discussion

In this study, we determined that the pre-application of LPS inhibits ATP-evoked currents, calcium influx, and delays macropore formation in P2X7R-HEK293 cells without modifying ERK activation. In the absence of ATP, LPS is unable to induce any response related to P2X7R activation, suggesting that LPS acts as a receptor modulator. Additionally, we observed inhibition of ATP-induced apoptosis in regulatory T cells by LPS treatment, effect that was reversed by a P2X7R antagonist, suggesting that this phenomenon could be of physiological relevance. 

Using different experimental approaches we consistently found that LPS modulates processes related to the channel activity of the P2X7R but not to ERK activation, a process that has been proposed to be independent of P2X7R channel activity. Ampstrup and Novak described that in P2X7R-expressing but not in control HEK293, ATP is able to induce ERK activation [31]. Moreover, this effect is preserved in cells expressing mutated receptors that do not exhibit channel activity, suggesting that P2X7R can signal through multiple pathways [31]. Accordingly, Garcia-Marcos et al. described the existence of 2 receptor pools involved independently in the P2X7R channel or signaling activity [32, 33]. These authors suggested that lipid interactions with the P2X7R on plasma membrane raft domains are important in defining its downstream response. It appears that receptors present on rafts are exclusive for signal transduction (like ERK phosphorylation), while nonraft P2X7Rs act mainly as ionic channels (reviewed in [33]). In this context, it is possible that LPS induces a relocalization of the P2X7R, decreasing the pool of receptor's able to be activated as channels.

The specific mechanism of LPS-induced inhibition of P2X7R channel activity remains unclear; however, some plausible explanations consider the putative C-terminal LPS-binding site (residues 573–590), originally described by Denlinger et al. [20, 34]. This domain appears to be highly functional given that peptides derived from this sequence are able to bind LPS *in vitro* and inhibit LPS-induced ERK activation in RAW 264.7 cells [20, 34]. It has been suggested that this phenotype correlates with changes in receptor trafficking, impairing P2X7R expression in the plasma membrane. In this context, it is reasonable to assume that under our experimental conditions, LPS is binding to its putative site in the P2X7R, interfering with the normal trafficking and assemblage of the receptor in the cell surface. This mechanism could explain the gradual decrease in currents and calcium transients as well as the inhibition of BrEt uptake that we observed after LPS application to P2X7R-HEK293 cells. Ongoing experiments in our lab will address this possibility in future works.

Although a direct interaction of LPS with the P2X7R seems the most reasonable mechanism, it is also possible that LPS effects are indirect and mediated by proteins other than TLRs [35]. These putative targets include the activation of the inflammasome (in codependence of P2Xs and TLRs activation [36]) and could be related to the P2X7R-pannexin-1 complex, caspase activation, and production of inflammatory mediators [37, 38]. Ectonucleotidase inhibition by LPS [39–41] does not seem to be involved under our experimental conditions; it has been demonstrated that ATP degradation appears to be lower and not significant [14]. The possibility cannot be excluded that LPS could interact directly with ATP, decreasing its free concentration. However, there is extensive evidence that lipids can modulate the P2X7R, either positively or negatively (as allosteric potentiators or inhibitors), suggesting that these molecules could interact directly with receptor regions and thereby exert their modulatory effect [13–16]. It would be interesting to determine how subtle modifications in lipid structure affect P2X7R. Future investigations will help to clarify this and determine structure-activity relationships, including possible studies with other LPS serotypes.

In our hands, LPS inhibited P2X7R activity when cells were pre-treated with LPS. In microglial cells, LPS had no significant effect on P2X7R-mediated currents although Raouf et al. observed a tendency for currents to decrease [42]. The apparent discrepancy between this study and our work could be explained by the fact that the electrophysiological recordings of Raouf et al. were performed in the absence of serum [42]. How LPS exerts its effects on cells is not clear. LPS has a complex pattern to induce cell activation, including several pathways for cell entry. Its internalization, which is energy-dependent [43], includes clathrin-coated vesicles [44–48], macropinocytosis [49], noncoated plasma membrane invaginations [43], and also occurs by nonspecific endocytosis. Once inside the cell, LPS has been detected in endosomes, phagosomes, lysosomes, the golgi complex [44–48], and the cytoplasm [50, 51]. However, the mechanism by which LPS passes through the plasma membrane of the cell is unknown. 

In our study, we consistently found that FBS increases the amplitude of ATP-induced currents. Although we did not study this point in detail, we can infer that serum components, such as cytokines, growth factor, and other proteins are responsible for this effect. Previously, Michel et al. reported that serum, and specifically albumin, could affect the affinity of P2X7R for BzATP, measured as ethidium bromide uptake [14]. Recently 2 immune compounds have been identified as P2X7R activators. Serum amyloid protein (SAA) can induce the expression of pro-IL-1*β* and activation of the NLRP3 inflammasome via P2X7R [52]. Similarly the human cathelicidin-derived peptide LL37 induces maturation and release of IL-1*β* via the P2X7R in LPS-primed monocytes [53]. These compounds could be present in the serum and potentially modify P2X7R activity. Another possibility is the capacity for serum to chelate divalent cations, which are known to inhibit the P2X7R. It would be highly relevant to identify the serum component(s) responsible for the effects on the P2X7R and determine the mechanism that affects receptor activity.

The role of the P2X7R in regulatory mechanisms associated with inflammation is well established. Ferrari et al. were the first to demonstrate that ATP potentiates post-translational processing and the release of IL-1*β* from LPS primed cells and that the P2X7R plays an important role in this process [54]. This has further been supported by the fact that P2X7R KO mice show a loss of ATP-dependent immune functions [7, 55]. Moreover, P2X7R KO mice have increased numbers of Foxp3-expressing CD4+ CD25+ cells in the circulatory and lymphoid organs, indicating that P2X7Rs may play a role in T-cell homeostasis [6, 56]. In this regard, it has been demonstrated that P2X7R activation leads to rapid cell death in a large proportion of the CD4+ CD25+ population, without significantly affecting other T cell populations [24]. In their study, Perregaux and Gabel argued that these dramatic differences in sensitivity among T cells appear to be related to the presence of the P2X7R [57], in agreement with our results. Gene array analyses indicate higher expression of P2X7Rs in Treg cells compared to conventional T-effector cells [58] and cell sensitivity in cells, and mouse strain shows a correlation with P2X7R surface expression [59]. In this context, large amounts of ATP can be highly deleterious, leading to the induction of apoptosis, impairing inflammatory and immune response. Given that P2X7R ligands can also influence LPS-stimulated IL-*β* release, ion fluxes, phospholipase and transcription factor activation, protein kinase cascades, and apoptosis, the regulation among those activities should be fine-tuned. 

In summary, our results provide evidence for the physiological relevance of LPS as a modulator of the P2X7R through its putative intracellular binding site. This process could be relevant during inflammatory or infectious processes, where extracellular ATP availability is high and/or maintained over time. The presence of LPS under these conditions could favor signal transduction instead of channel activation and apoptosis, leading to a more efficient response against pathogens.

## Supplementary Material

Sup. Fig. 1: HEK293 cells do not express the TLR-4receptor. PCR were done for TLR4 and RNAr 16S has a housekeeping gene. Reactions obtained were electrophorized in agarosa gel and visualized by UV staining in transilluminator.
Sup. Fig. 2: P2Y have lower contribution in ATP inducing calcium movement. Representative recordings showing wild-type (upper panel) and P2X7R-expressing (lower panel) HEK293 cells. In the complete absence of extracellular calcium (by the addition of 5 mM EGTA), no responses were observed. These results were observed in at least 3 different cells.
Sup. Fig. 3: LPS alone cannot trigger ERK activation in P2X7R-HEK293 cells. (A) Representative gel showing ERK activation is induced by 1 mM ATP and the lack of effect of LPS alone (1 *µ*g/mL) applied at different times. (B) LPS-induced activation of ERK in Raw 264.7 cells; these cells express the classical LPS receptor TLR-4. In both cases upper panels show the detection of P-ERK and lower panels show total ERK.
