# Supplementary material for: Lipopolysaccharide Inhibits the Channel Activity of the P2X7 Receptor
Source: Mediators Inflamm. 2011 Sep 14;2011:152625. doi: 10.1155/2011/152625 (PMC3173735; doi:10.1155/2011/152625)

Sup. Fig. 1

Hek 293 cells does not express Tlr4receptor. PCR were done for TLR4 and RNAr 16S has a housekeeping gene. Reactions obtained were electrophorized in agarosa gel and visualized by UV staining in transilluminator.

Sup. Fig. 2

P2Y have lower contribution in ATP inducing calcium movement. Representative recordings showing wild-type (upper panel) and P2X7R-expressing (lower panel) HEK293 cells. In the complete absence of extracellular calcium (by the addition of 5 mM EGTA), no responses were observed. These results were observed in at least 3 different cells.

Sup. Fig. 3

LPS alone cannot trigger ERK activation in P2X7R-HEK293 cells. (A) Representative gel showing ERK activation is induced by 1 mM ATP and the lack of effect of LPS alone (1 µg/mL) applied at different times. **(B)**. LPS-induced activation of ERK in Raw 264.7 cells; these cells express the classical LPS receptor TLR-4. In both cases upper panels show the detection of P-ERK and lower panels show total ERK.

Supplemental figure 1

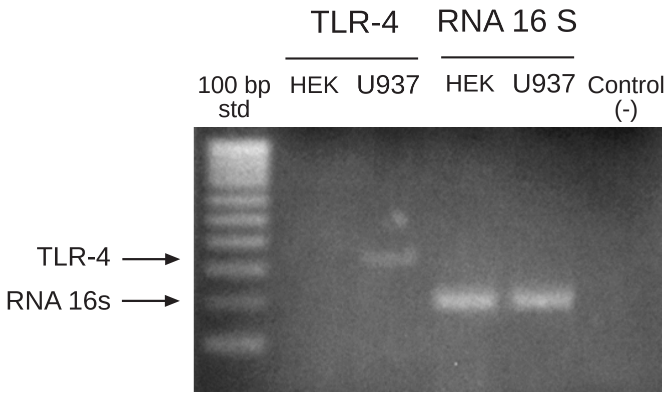

Supplemental figure 2

HEK293

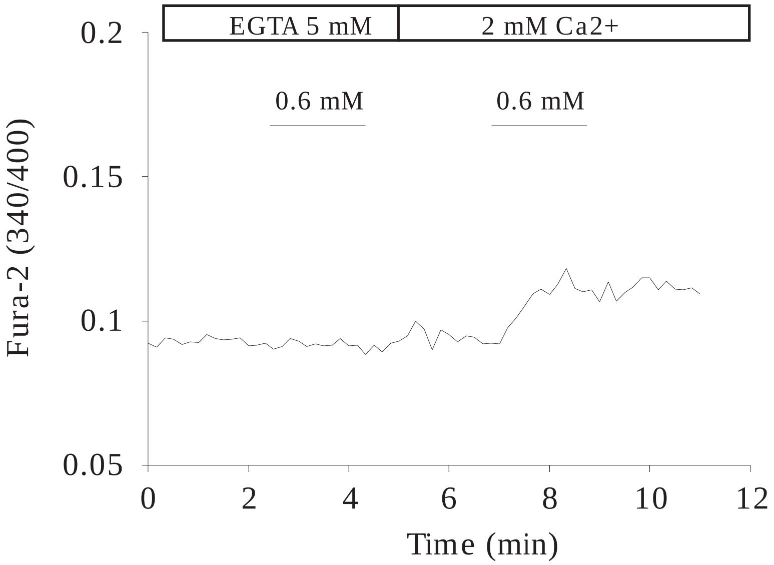

HEK293-P2X7R

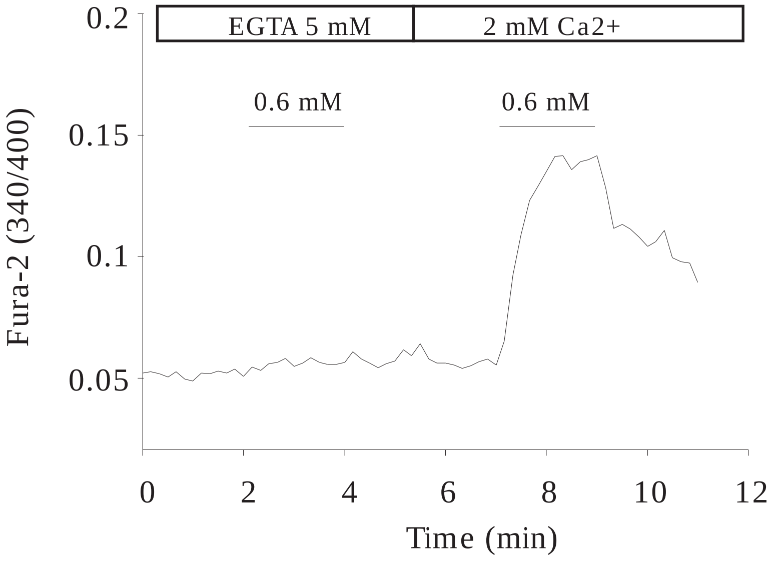

Supplemental figure 3

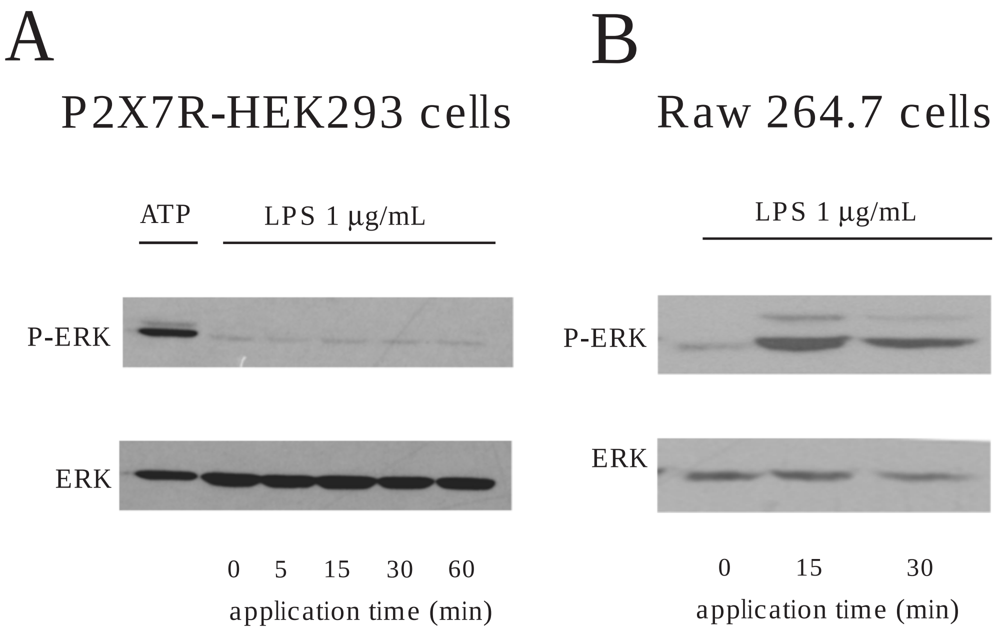

Supplement: Supplementary file 1 — Sup. Fig. 1: HEK293 cells do not express the TLR-4receptor. PCR were done for TLR4 and RNAr 16S has a housekeeping gene. Reactions obtained were electrophorized in agarosa gel and visualized by UV staining in transilluminator. Sup. Fig. 2: P2Y have lower contribution in ATP inducing calcium movement. Representative recordings showing wild-type (upper panel) and P2X7R-expressing (lower panel) HEK293 cells. In the complete absence of extracellular calcium (by the addition of 5 mM EGTA), no responses were observed. These results were observed in at least 3 different cells. Sup. Fig. 3: LPS alone cannot trigger ERK activation in P2X7R-HEK293 cells. (A) Representative gel showing ERK activation is induced by 1 mM ATP and the lack of effect of LPS alone (1 µg/mL) applied at different times. (B) LPS-induced activation of ERK in Raw 264.7 cells; these cells express the classical LPS receptor TLR-4. In both cases upper panels show the detection of P-ERK and lower panels show total ERK. [file 152625.f1.pdf]
