# Supplementary material for: Lipopolysaccharide Inhibits the Channel Activity of the P2X7 Receptor
Source: Mediators Inflamm. 2011 Sep 14;2011:152625. doi: 10.1155/2011/152625 (PMC3173735; doi:10.1155/2011/152625)
Supplement: Supplementary file 2 [file 152625.f2.pdf]

Calcium determination

For Fura-2 AM, transfected or control HEK293 cells were seeded at 105 cells on 35mm coverslips, mounted in a perfusion chamber on the stage of an inverted microscope (Olympus IX-81, UPLFLN 40XO 40x/1.3 oilimmersion objective). Cells were incubated with 4 µM Fura-2 AM (Molecular Probes) for 20min. The bath solution without calcium contained (mM) 140NaCl, 5 KCl, 5 EGTA, 1MgCl2, 10 glucose, and 10 HEPES, pH 7.4, adjusted with HCl. The bath solution with calcium contained (mM): 140NaCl, 5 KCl, 2 CaCl2, 1MgCl2, 10 glucose and 10HEPES, pH 7.4. ATP was applied by changing the specific bath solution (without or with calcium) with the respective ATP concentration. Fura-2 was alternately excited at 340 and 400 nm, and the fluuorescence filtered at 510nm was collected and recorded each 10 s using a CCD-based imaging system (Olympus DSU) running CellR software (Olympus). For every experiment, signals were recorded, and the background intensity was subtracted, using a same-size region of interest outside the cells. Results are expressed as the ratio between the 340nm and 400nm (R340/400) signals.

RNA extraction and PCR

RNA extraction and RT-PCR and visualization of PCR products were done as previously described [23]. Amplification of 16S was done using specific primers (sense: 5_-GGGGTTTACGACCTCGATGTT-3_ antisense: 5_-GCTTTAAGTATGGGCCCCCCT-3_); PCR conditions were 94ºC for 4min followed by 29 cycles of 94ºC for 40 s, 50ºC for 40 s, and 72ºC for 20 s. TLR-4 amplification was done using specific primers (sense CAACAAAGGTGGGAATGCTT 317, antisense TGCCATTGAAAGCAACTCTG), with PCR conditions 94ºC for 4min followed by 30 cycles of 98ºC for 10 s, 60ºC for 2min, 74ºC for 15 s.

Western blots

Raw 264.7 cells or HEK 293T cells (3 x 106 cells) were seeded in six-well plates (Nunc, Roskilde, Denmark). After 12 h, cells were stimulated for various times with LPS 100 ng/ml (serotype 055:B5, Sigma Chemical Co, St. Louis MO, USA). The medium was removed, cells were washed twice with cold phosphate buffer saline, scraped and lysed with Laemmli buffer containing 5% b-mercaptoethanol at 80°C. Total cell extracts were frozen at –20ºC until needed. Western blots were done under standard conditions, and MAPKs activity was assessed by reactivity against anti active-ERK, anti- pan ERK (Cell Signaling Technology, Beverly, MA, USA), and actin (ICN Biomedicals, Inc., Irvine, CA, USA) antibodies, according to the instructions of the manufacturers. Membranes were then re-probed with anti- pan ERK (Transduction Laboratories), anti active ERK (Cell Signaling), and actin (ICN) antibodies. Immunoreactive bands were visualized using the ECL enhanced chemiluminescence detection system (Amersham, Arlington Heights, IL, USA) by film exposure (Kodak Biomax Light Film, Rochester, NY, USA).
